# Biopolymers from lactic acid bacteria. Novel applications in foods and beverages

**DOI:** 10.3389/fmicb.2015.00834

**Published:** 2015-09-11

**Authors:** María I. Torino, Graciela Font de Valdez, Fernanda Mozzi

**Affiliations:** Technology Department, Centro de Referencia para Lactobacilos – Consejo Nacional de Investigaciones Científicas y Técnicas, San Miguel de TucumánArgentina

**Keywords:** exopolysaccharides, homopolysaccharides, heteropolysaccharides, lactic acid bacteria, fermented foods, fermented beverages, sourdough

## Abstract

Lactic acid bacteria (LAB) are microorganisms widely used in the fermented food industry worldwide. Certain LAB are able to produce exopolysaccharides (EPS) either attached to the cell wall (capsular EPS) or released to the extracellular environment (EPS). According to their composition, LAB may synthesize heteropolysaccharides or homopolysaccharides. A wide diversity of EPS are produced by LAB concerning their monomer composition, molecular mass, and structure. Although EPS-producing LAB strains have been traditionally applied in the manufacture of dairy products such as fermented milks and yogurts, their use in the elaboration of low-fat cheeses, diverse type of sourdough breads, and certain beverages are some of the novel applications of these polymers. This work aims to collect the most relevant issues of the former reviews concerning the monomer composition, structure, and yields and biosynthetic enzymes of EPS from LAB; to describe the recently characterized EPS and to present the application of both EPS-producing strains and their polymers in the fermented (specifically beverages and cereal-based) food industry.

## Introduction

Since ancient times lactic acid bacteria (LAB) have been empirically exploited as starter cultures to improve the preservation, nutritional value, and sensorial characteristics of a variety of fermented foods and products derived from animal and vegetable origins (Wood and Holzapfel, 1995; Wood, 1997; Leroy and De Vuyst, 2004). LAB have been rationally applied as lactic starter cultures in the fermented food industry since the 1930s–1940s. In addition, certain LAB strains have been used as probiotics because of their health-promoting effects in the host (Foligné et al., 2013; Martín et al., 2013; Borges et al., 2014). Due to their long history of safe use in human consumption, some LAB strains have the Qualified Presumption of Safety (QPS) or Generally Recognized As Safe (GRAS) status (EFSA, 2010).

In addition to their main feature, which is lactic acid production from the carbon source present in the matrix where they grow, several LAB strains form other compounds such as vitamins, bioactive peptides, antibacterial compounds, aroma compounds, low-calorie sugars, exopolysaccharides (EPS), etc. All these traits confer desirable attributes to specific fermented foods and products (Hugenholtz, 2008; LeBlanc et al., 2013; Ortiz et al., 2013). In this respect, efforts have been made to use LAB as microbial cell factories for the production of industrially interesting metabolites either to be used as purified compounds or to be produced *in situ* in fermented foods (Hugenholtz, 2008; Gaspar et al., 2013; Boguta et al., 2014).

In this work, we aimed to collect the most relevant issues of former reviews on EPS from LAB such as their monomer composition, structure, yields and biosynthetic enzymes; to describe the recently characterized EPS and to present the application of both EPS-producing strains and their polymers in the fermented (specifically beverages and cereal-based) food industry.

Several reviews on EPS produced by LAB have been published dealing mainly with the physiology, biosynthesis, chemical and structural characteristics of the EPS molecules. To our criteria, the most relevant and detailed works covering these topics through the last decade include those from Ruas-Madiedo and de los Reyes-Gavilán (2005), Badel et al. (2011), Patel et al. (2012), Leemhuis et al. (2013b). Regarding the health-promoting benefits of these polymers two reviews on their immunomodulatory activity and prebiotic effects were recently addressed by Ryan et al. (2015) and Salazar et al. (2015).

## EPS Classification, Biosynthesis, and Yields

As the majority of bacteria, LAB can synthesize cell-wall structural polysaccharides such as peptidoglycan and lipoteichoic acids, and exocellular polymers. The latter include both capsular polysaccharides (CPS), covalently bound to the cell surface, and EPS, which may form a loosely bound layer that can also be secreted into the environment (Chapot-Chartier et al., 2011).

Exopolysaccharides from LAB are highly diverse and can be classified following different criteria. The most classical one is based on their monomer composition, which allows classifying them into two major groups: homopolysaccharides (HoPS) and heteropolysaccharides (HePS).

### Homopolysaccharides

The most notorious advance in the research on EPS from LAB in the last decade has been related to HoPS. Indeed, the isolation of HoPS-producing strains (mainly belonging to the *Weissella* genera), the molecular and structural characterization of these EPS, studies on their biosynthetic enzymes, and the HoPS application in food have been described (Galle and Arendt, 2014; Lynch et al., 2014; Tingirikari et al., 2014; Wolter et al., 2014a,b).

Homopolysaccharides contain one neutral monosaccharide type either glucose (glucans), fructose (fructans), or galactose (polygalactan) (Monsan et al., 2001; Mozzi et al., 2006; Ruas-Madiedo et al., 2008). Among LAB, a sub-classification has been established depending on the linkage type and the position of the carbon involved in the bond. Thus, glucans can be sub-classified into (i) α-glucans [dextran: α-D-Glc(1,4); mutan: α-D-Glc(1,3); alternan: (α-D-Glc(1,6)/α-D-Glc(1,3); and reuteran: α-D-Glc(1,4)/α-D-Glc(1,6) with α-D-Glc(1,4)/α-D-Glc(1,6) branching points], and (ii) β-glucans [β-D-Glc(1,3) with side chain linked (1,2)]. Fructans can be classified into (i) levan-type: β-D-Fru(2,6), and (ii) inulin-type: β-D-Fru(2,1), being both β-fructans. Finally, polygalactans, which contain a pentameric repeating unit of galactose; these polymers being more rare and were only described for the strain *Lactococcus. lactis* subsp. *lactis* H414 (van Kranenburg et al., 1999) and two strains of *Lactobacillus delbrueckii* subsp. *bulgaricus* (CRL 406 and 142; Mozzi et al., 2006). Recently, a polygalactan-containing CPS from the strain *Lactobacillus plantarum* 70810 was analyzed by different techniques (GC-MS, gas chromatography–mass spectrometry; FTIR, fourier transform infrared spectroscopy; and NMR, nuclear magnetic resonance) resulting in a low molecular weight (MW) polymer of 1.7 kDa with a repeating unit of α-D-(1,6)-linked galactcosyl, β-D-(1,4)-linked galactcosyl, β-D-(1;2,3)-linked galactcosyl residues and a tail end of β-D (1,)-linked galactcosyl residue (Wang et al., 2014).

Traditional fermented foods can be a rich source of HoPS-producing LAB as well as of different polymers. Recently, several dextran-producing LAB strains from sourdoughs and indigenous Asian foods were isolated (**Table 1**).

**Table 1:** Dextran-producing LAB strains and properties of their polymers.

Microorganisms	Source	Dextran characteristics^&^	Structural studies*	Molecular studies	Reference
*Leuconostoc mesenteroides* NRRL B-1149	Sugar-cane juice	Insoluble (medium used: sucrose, 10.0%; maltose, 5.0%; 20 mM sodium acetate buffer (pH = 5.4) containing 0.1% sodium azide and purified dextransucrase (0.2 mg/mL, 13 U/mg) Fibrous structure	FTIR, ^1^H NMR, ^13^C NMR spectroscopy and SEM technique	Purification and characterization of dextransucrase (enzyme activity in SDS-PAGE)	Shukla et al., 2010, 2011
*Pediococcus pentosaceous* CRAG 3	Fermented cucumber	MM 2.9 × 10^5^ Porous structure	FTIR, NMR, SEM	Purification and characterization of dextransucrase (SDS-PAGE)	Shukla and Goyal, 2013, 2014
*P. acidilactici* M76	Korean fermented rice wine (*makgeolli*)	MM 6.7 × 10^4^	SEC, FTIR		Song et al., 2013
*Lactobacillus hilgardii* TMW 1.828	Water kefir			Purification and sequence analysis of glucansucrase (GS). Characterization of GS activity in SDS-PAGE. Identification and sequence of glucansucrase gene [FN662554]	Waldherr et al., 2010
*L. acidophilus* ST76480.01	Fermented vegetables	Insoluble (medium used %: sucrose, 15.0; bacto-peptone, 0.5; yeast extract, 0.5; K_2_HPO_4_, 1.5; MnCl_2_.H_2_O, 0.001; NaCl, 0.001; CaCl_2_, 0.005 and pH 7.0)	Rheology, ^13^C-NMR spectroscopy.	Characterization of dextransucrase activity (SDS-PAGE)	Abedin et al., 2013
*L. plantarum* DM5	Indian traditional fermented beverage (*Marcha*)	MM 1.1 × 10^6^ Smooth porous structure	SEC, FTIR, NMR, SEM, rheology	Purification and characterization of GS (SDS-PAGE)	Das and Goyal, 2014
*Weissella confusa* MBF8-1	Indonesian fermented soybean foods (*Pamulang, Tangerang*)			Two *gtf* genes: *gtf8-1A* [FJ436354] and *gtf8-1B* [FJ460018]	Malik et al., 2009
*Weisella.* sp. TN610	Pear		NMR	Characterization of dextransucrase activity (SDS-PAGE). Sequence of *gtf* gene [HE818409]	Bejar et al., 2013
*W. cibaria* MG1	Sourdough	3 x 10^9^, 5 x 10^6^–4 x 10^7^ GOS is formed in addition to dextran when maltose is present	FFF, SEC, HPAEC-PAD	Genome sequence including the *gtf* gene (JWHU00000000)	Galle et al., 2010, 2012a,b; Zannini et al., 2013; Lynch et al., 2014; Wolter et al., 2014a,b
*W. cibaria* JAG8	Apple peel	Porous structure	DLS spectroscopy (monodisperse), SEM	Purification of dextransucrase; *in vitro* synthesis of dextran	Rao and Goyal, 2013

*Information published by Ruas-Madiedo and de los Reyes-Gavilán (2005) and Badel et al. (2011) was omitted in this Table to avoid overlapping.*

^&^MM, molecular mass expressed in Daltons; GOS, gluco-oligosaccharides.

*FITR, fourier transform infrared; ^1^H NMR and ^13^C NMR, nuclear magnetic resonance spectroscopic; SEM, scanning electron microscopy; SEC, size-exclusion chromatography; MALLS, multiangle laser light scattering; HPAEC-PAD: high performance anion exchange chromatography with pulsed amperometric detection; DLS, dynamic light scattering.

In addition Park et al. (2013) have isolated dextran-like polymer-producing strains belonging to the genera *Leuconostoc* and *Weissella* from kimchi. The MW of these HoPS being about 1.1 × 10^6^ Da; FTIR analysis showed that these polymers had a similar structure to that of commercial dextran from *Leuc. mesenteroides* B-512F. *Leuconostoc* strains produce putative alternan polymers being those α-(1,2) branched polymers particularly high (Passerini et al., 2015). Interestingly, the ^1^H NMR spectra of the EPS produced by *L. curvatus* 69B2 and *Leuc. lactis* 95A showed that the HoPS formed was constituted by a single repeating glucopyranosyl unit linked by an α-(1,6) glycosidic bond in a dextran-type carbohydrate (Palomba et al., 2012).

Patel et al. (2012) showed that some strains of *L. fermentum*, *L. sakei*, and *L. hilgardii* produced α-(1,6) glucans ramified by glucose residues at position 3, and at lesser extent at positions 2 and 4 (dextran). The degree of branching involving α-1,2, α-1,3 and α-1,4 linkages varying according to the origin of the biosynthetic enzymes.

Some species such *L. reuteri*, a predominant HoPS producer, is also able to generate different types of EPS including dextran (Rühmkorf et al., 2013), levan, inulin-type fructan, mutan, and reuteran with a broad MW range. Also, the single strain *L. reuteri* 121 synthesizes several HoPS when grown under the same culture conditions (van Geel-Schutten et al., 1999; van Leeuwen et al., 2009; Pijning et al., 2012).

Respect to HoPS biosynthesis, these polymers are mainly synthesized extracellularly from an existing sucrose molecule, which acts as donor of the corresponding monosaccharide by action of a single type of extracellular enzyme belonging to the glycosyl hydrolase (GH) family. Thus, α-glucans and β-fructans are formed by glucansucrases (GS; GH family 70) and fructansucrases (FS; GH family 68), respectively (http://www.cazy.org; Cantarel et al., 2009). Generally, these enzymes catalyze the polymerization of the HoPS out of sucrose as donor of the corresponding monosaccharide and transfer the molecule to the reducing end of the glucan or fructan, respectively (Korakli and Vogel, 2006; van Hijum et al., 2006; Chapot-Chartier et al., 2011; Leemhuis et al., 2012). Both enzymes are typical transglycosylases or glycansucrases displaying a dual mode of action as they can cleave the glycosidic bond in sucrose (hydrolysis reaction) and with the energy released they can transfer the glucosyl or fructosyl moiety (transferase reaction) to the growing reducing end of the polymer (Chapot-Chartier et al., 2011).

Glucansucrases can synthesize a variety of α-glucans with different physicochemical characteristics such as solubility, viscosity, and other properties by altering the type of glycosidic linkage, degree of branching, length, mass, and conformation of the polymers. So far, α-glucan formation by GS has been reported for the genera *Leuconostoc, Lactobacillus, Streptococcus, Pediococcus*, and *Weissella*. Depending on the reaction catalyzed and the specificity, GS are classified by the Nomenclature Committee of the International Union of Biochemistry and Molecular Biology (NC-IUBMB) in four groups: dextransucrase (EC 2.4.1.5), mutansucrase (EC 2.4.1.5), alternansucrase (EC 2.4.1.140), and reuteransucrase (EC 2.4.1.5), which catalyze α-(1,6), α-(1,3), α-(1,3 and 1,6), and α-(1,4 and 1,6) glycosidic linkages, respectively (Leemhuis et al., 2013b).

The *in vitro* synthesis of glucooligosaccharides (GOS) and fructooligosaccharides (FOS) by using selected GS and FS and electron acceptors reaction (i.e., maltose, raffinose) was reported in *Lactobacillus* (Tieking et al., 2005a; Ozimek et al., 2006; Katina et al., 2009; Galle et al., 2010, 2012b; Shukla and Goyal, 2014), *Leuconostoc* (Leemhuis et al., 2013a), and *Weissella* (Malang et al., 2015). This approach constitutes one of the most interesting perspectives to design short tailor-made HoPS with prebiotic activity.

Glucansucrases expression is involved in the constitutive production of HoPS as reported in the strains *L. reuteri* 121, 180 and *L. parabuchneri* 33. On the contrary, the GS expression by *Leuc. mesenteroides* is induced by sucrose (Kralj et al., 2004), these enzymes are high MW extracellular proteins exclusively encoded by GS (*gtf*) genes. Variations in EPS structures, even belonging to the same group (i.e., dextran, mutan, etc.) are due to different enzymes intervention. For example, the product of the gene *gtf* Kg3 from *L. fermentum* Kg3 is responsible for the synthesis of a dextran which is 89% linked by α-(1,6) linkages, while *gtf* 180 (from *L. reuteri* 180) encoded for a protein involved in the synthesis of a dextran with 51% of α-(1,6) glucosidic bonds. In addition, biosynthesis of glucans by a wine strain of *Lactobacillus* sp. leads to a linear dextran whereas *L. reuteri* 180 synthesizes a branched EPS (Uzochukwu et al., 2001; van Leeuwen et al., 2009). Branching is catalyzed by GS involved in the HoPS backbone synthesis; the degree of branching depending on the enzyme conformation. Some experiments accomplished on GS from the strain *L. reuteri* 121 showed that mutations of the gene encoding for this enzyme increased α-(1,6) and decreased α-(1,4) linkages.

The production of β-glucan has mainly been described in LAB isolated from alcoholic fermented beverages and its synthesis occurs intracellularly by a membrane-associated glucosyltransferase (GTF). Although its mechanism of action is not fully yet understood, it does not need sucrose as substrate (Werning et al., 2006). LAB strains belonging to the genera *Pediococcus*, *Lactobacillus*, and *Oenococcus* isolated from cider and wine are able to produce a 2-substituted (1, 3)-β-D-glucan (Llaubères et al., 1990; Dueñas-Chasco et al., 1997, 1998; Ibarburu et al., 2007; Dols-Lafargue et al., 2008) by a single transmembrane GTF that polymerizes glucosyl residues from UDP-glucose (Werning et al., 2006, 2008; Velasco et al., 2007; Garai-Ibabe et al., 2010). Considering the requirement of sugar nucleotide intermediates as precursors, the synthesis of β-glucans resembles HePS’s synthesis and it is linked to the growth and the central carbon metabolism of the producer organism. Also, GTF are part of the enzyme machinery responsible for HePS formation (De Vuyst and Degeest, 1999). The (1,3)-β-D-glucans are attractive for the pharmaceutical and functional food industries because of their beneficial effects on human and animal health (Zekovic et al., 2005). The 2-substituted (1,3)-β-D-glucan producers *P. parvulus* 2.6 and *L. paracasei* NFBC 338 have been tested for the production of oat-based products, yogurt and various beverages increasing significantly their techno-functional properties (Mårtensson et al., 2002; Kearney et al., 2011). Moreover, the synthesis of the 2.6 β-glucan confers higher survival to the producing-strain during the gastrointestinal passage or technological process (Stack et al., 2010).

The enzyme FS or fructosyltransferase (FS, EC 2.4.1.10) cleaves the glycosidic bond of the fructosyl-donor molecule (substrate, i.e., sucrose, raffinose, stachyose, verbascose) and uses the released energy to couple a fructose moiety to a growing fructan chain but also to sucrose or to another acceptor such as raffinose (Meng and Fütterer, 2003, 2008; van Hijum et al., 2006; Teixeira et al., 2012). As mentioned earlier, two fructan types are known, levan [with β-2,6 glycosidic bonds, produced by levansucrases (Lev, E.C. 2.4.1.10; van Hijum et al., 2004)] and inulin (with β-2,1 bonds) synthesized by inulosucrase (Inu, E.C. 2.4.1.9; van Hijum et al., 2003, 2006). Fructan production has been reported for *W. confusa* strains isolated from Malaysian soy, Malaysian coconut milk beverage (Malik et al., 2009), wheat sourdough (Tieking et al., 2003), fermented cassava (Malang et al., 2015), and for *L. reuteri* Lb121 (van Geel-Schutten et al., 1999) and a *L. pontis* strain (Tieking et al., 2003). Inulin biosynthesis is rare in LAB and only individual strains of *Lactobacillus* and *Leuconostoc* as well as a few streptococci were reported to produce inulin or to possess inulosucrase-encoding genes (van Hijum et al., 2002; Olivares-Illana et al., 2003; Schwab et al., 2007; Anwar et al., 2008, 2010). Recently, Malang et al. (2015) reported the first structural characterization of a fructan-type polymer produced by a *Weissella* strain as well as the first *Weissella* strain to produce inulin in addition to oligosaccharides. By means of NMR, the soluble EPS formed by nine strains were identified as low α-1,3-branched dextran, levan and inulin-type polymers. Besides, six isolates synthesized a highly ropy polymer together with CPS formation composed of glucose, *O*-acetyl groups and two unidentified monomers when using glucose as carbon source.

The fact that GTF cannot use sucrose as an acceptor but GS can, and that FS can also use raffinose, stachyose, and verbascose as substrates, are clear differences among these enzymes. Indeed, the reactions formed by FS and GS are similar with respect to the use of sucrose as a substrate (Galle and Arendt, 2014), but the proteins involved do not share a high amino acid sequence similarity and differ strongly in protein structures (Vasileva et al., 2009; Leemhuis et al., 2013b).

Due to the interest in the food industry, the number of isolations of GS-producing LAB has been rapidly increasing. The strains are typically identified on solid or liquid media supplemented with sucrose by the appearance of slimy/ropy colonies or viscous solutions. Gene knock-out studies have demonstrated that the slimy colony morphology is caused by GS activity in wild-type strains (Fujiwara et al., 2000; Waldherr et al., 2010). However, also β-fructan-producing strains form slimy colonies in the presence of sucrose. Replacing sucrose by the tri-saccharide raffinose used by FS but not by GS enzymes, allows easy identification of β-fructan-producing strains. By this means, many α–glucan-(and β-fructan-)-forming strains have been identified in fermenting cabbage, fruits, and vegetables, sourdoughs, beverages, dairy, cereals, dental plaque, intestines, environmental spills of sugar plants, and in fish gastrointestinal tract (van Geel-Schutten et al., 1998; Tieking et al., 2005b; Van der Meulen et al., 2007; Kang et al., 2009; Malik et al., 2009; Bounaix et al., 2010a,b; Shukla and Goyal, 2011; Aman et al., 2012; Hongpattarakere et al., 2012). Nevertheless, the number of well-characterized GS remains behind with respect to available *gtf* sequences as only three-dimensional structures of GS complexes (from *Leuc. mesenteroides* NRRL B-1299, *Streptococcus mutans* and *L. reuteri* 180) are available (Vujičić-Žagar et al., 2010; Ito et al., 2011; Brison et al., 2012). Expanding the structural knowledge to a wider range of GS and FS, either alone or complexed with different molecules (substrates or products) may pave the way for the rational application of HoPS. Specific information on the structure-function relationships in GS- and FS-producing HoPS, and on their structures, reactions, and GS mechanisms has been reported by van Hijum et al. (2006) and Leemhuis et al. (2013a), respectively. The GS from strains *L. reuteri* 121 and TMW 1.106, *L. curvatus* TMW 1.624, *L. animalis* TMW 1.971, and *L. hilgardii* TMW 1.828 were biochemically characterized (Kralj et al., 2004; Waldherr et al., 2010; Rühmkorf et al., 2013).

The yields of HoPS produced by LAB are low as compared with other bacterial EPS (i.e., xanthan from *Xanthomonas campestris*) already used in the food industry. It has been reported that EPS formation by *Weisella* sp. and *L. sanfranciscenis* strains reached levels up to 16 and 5 g EPS/kg dough, respectively, thus showing their potential to replace hydrocolloids (Galle and Arendt, 2014). Also, HoPS production values up to 10 g/L by the strain *L. reuteri* Lb121, which simultaneously synthesizes α-glucan and β-fructan has been reported (van Geel-Schutten et al., 1999). The MW of HoPS varies according to the producing-strain (Ruas-Madiedo et al., 2009a,b). The average MW of soluble dextran is in a range between 6.2 and 7.1 × 10^6^ Da, bacterial inulins’ MW range between 1 and 9 × 10^7^ g/mol (Shiroza and Kuramitsu, 1988; van Hijum et al., 2002), while the MW of levans are within the range of 10^5^–2 × 10^6^ g/mol for lactobacilli and *Leuconostoc* sp. strains and 10^8^ g/mol for *S. salivarius* (Tieking et al., 2003). Of note, the MW of *Weissella* levans (1.4 × 10^5^–1.7 × 10^7^ g/mol) depend on whether the strains grow on sucrose or raffinose as carbon source (Malang et al., 2015). The most remarkable difference among HoPS, specially inulin-like fructan and FOS or inulin is precisely the size, which directly correlates to their degree of polymerization (DP); FOS and inulin are short oligosaccharides (DP < 30, MW ~5 kDa) while HoPS are branched polymers of high MW (~1,000 kDa; Salazar et al., 2015).

### Heteropolysaccharides

Heteropolysaccharides from LAB were extensively studied at structural, genetic, and functional levels by Jolly and Stingele (2001) and Ruas-Madiedo and de los Reyes-Gavilán (2005).

In contrast to HoPS, HePS are complex polymers composed of a backbone of repeating subunits, branched or unbranched, that consist of three to eight monosaccharides, derivatives of monosaccharides or substituted monosaccharides. HePS usually contain D-glucose, D-galactose, and L-rhamnose although at different ratios. To a lesser extent, *N*-acetylated monosaccharides (*N*-acetyl-glucosamine and *N*-acetyl-galactosamine), other monosaccharides (fucose, ribose) as well as organic and inorganic (glucuronic acid, acetyl groups, glycerol, phosphate, etc.) substituents (De Vuyst and Degeest, 1999; De Vuyst et al., 2001; Mozzi et al., 2006) can be found in some HePS. Inversely to HoPS, the HePS reapeating units are intracellularly synthesized, and polymerized outside the cell. More than 45 different repeating units have been described by NMR spectroscopy (De Vuyst et al., 2001; Broadbent et al., 2003; Ruas-Madiedo et al., 2009a), mostly of these corresponding to EPS synthesized by LAB and a few by bifidobacteria strains (Ruas-Madiedo et al., 2012). In accordance with this chemical diversity the enzymatic machinery involved in the synthesis of HePS as well as the genes encoding these enzymes, which are organized in *eps* clusters showing an operon-like structure, are much more complex than those of HoPS (Jolly and Stingele, 2001; Broadbent et al., 2003; Ruas-Madiedo et al., 2009a). These genes are organized in four functional regions involved in chain length determination, polymerization, export, and regulation of the gene cluster (van Kranenburg et al., 1997). Because of their different compositions and MW, HePS may vary in charge, spatial arrangements, rigidity, and the ability to interact with proteins (Duboc and Mollet, 2001). All these characteristics strongly affect the EPS physico-chemical properties, such as solubility, viscosity, etc., (Monchois et al., 1999). Noticeable, a common property of the HePS from LAB is the high thickening power displayed by some of them even at very low concentrations (Vaningelgem et al., 2004). This fact seems to be linked to the molar mass (MM) of the EPS rather than the biopolymer charge as suggested by Mende et al. (2013). Indeed, when equal amounts of EPS or CPS from *S. thermophilus* ST-143 were added to milk prior to acidification (induced by a chemical acidulant), the stiffness of the acidified milk gels increased almost linearly with the EPS concentration whereas the CPS concentration did not affect the rheology of the acidified gels when the same amounts of CPS and EPS were added. Both EPS fractions were determined to be uncharged but differed distinctly in their MM, which was 2.6 × 10^6^ Da, and 1.4 × 10^5^ and 7 × 10^3^ Da for the EPS and CPS, respectively (the latter one being composed of two fractions). In contrast, Pachekrepapol et al. (2015) found that the EPS charge positively influenced the rheology of acidified milk gels when using dextran and dextran-sulfate.

Regarding the HePS MW a broad range, varying from 10^4^ to 10^6^ Da, has been reported. Interestingly, simultaneous occurrence of more than one chromatographic peak of different size in polymers synthesized by a given strain is often frequent in these HePS (Mozzi et al., 2006; Ruas-Madiedo et al., 2009a; Salazar et al., 2009). The EPS MW is a property of special relevance for the technological functionality of the polymers that strongly influence the viscosity and texture of the matrix in which they are present. An increased use of specific methodologies such as Multi-Angle Laser Light Scattering (MALLS) coupled to a refractometer and NMR have been applied to obtain concluding results on EPS MW and structure in the last years (Shao et al., 2015). By this way, Laws et al. (2008) confirmed the presence of EPS showing three (4, 1.5, and 1.3 × 10^5^ Da) and two (2.5 and 3.5 × 10^5^ Da) peaks isolated from cultures of *L. acidophilus* 5e2 growing in skim milk or skim milk plus glucose, respectively. Both HePS consist of a heptasaccharide repeating unit containing D-glucose, D-galactose and either *N*-acetyl-D-glucosamine, or D-glucosamine. Simultaneous occurrence of more than one chromatographic peak of different size in HePS synthesized by *L. rhamnosus* KL37B was also reported (Górska-Fraçzek et al., 2013b). In this case, different repeating units (penta- and nona-saccharide, respectively) were found for each peak, both containing D-glucose and D-galactose (1:2 molar ratio). Pentasaccharide repeating units are present in HePS from *L. johnsonii* 151 (1 × 10^5^ Da) and *L. helveticus* sp. *Rosyjski* (1 × 10^6^ Da) containing D-glucose and D-galactose (1:1.5 molar ratio) and D-glucose, D-galactose, and *N*-acetyl-D-mannosamine (2:2:1 molar ratio), respectively, (Górska-Fraçzek et al., 2013a; Patten et al., 2014).

In general, the HePS production levels reported for LAB are lower than those for HoPS. It has been observed that EPS-production is strain-dependent and is strongly affected by the microbial culture conditions such as the composition of the culture medium, pH, temperature, incubation time, etc., (Ruas-Madiedo and de los Reyes-Gavilán, 2005; Torino et al., 2005). Nevertheless, the use of different methods to isolate and quantify the HePS production makes difficult to compare yields accurately. To overcome this issue, methodologies for improving the purity of EPS and thus, to minimize variability in the methods were applied (Leemhuis et al., 2013b; Polak-Berecka et al., 2013). HePS yield values reported in the literature oscillate from 25 to 600 mg/L (Ruas-Madiedo et al., 2009b) while only a few strains have been reported to produce higher amounts under optimized growth conditions being the EPS yield (2 g/L) obtained with *L. rhamnosus* RW-9595M the highest reported to date (Bergmaier et al., 2005). Within the *S. thermophilus* species, Vaningelgem et al. (2004) reported HePS yields varying from 20 to 600 mg/L in milk-based medium under optimal culture conditions. Zisu and Shah (2003) reported the highest EPS production (1029 mg/L) for the strain *S. thermophilus* ASCC 1275 (ST 1275) in milk medium supplemented with 0.5% (w/v) whey protein concentrate (Zisu and Shah, 2003); this strain producing both CPS and ropy EPS (Zisu, 2005).

## Methodological Approaches

The structural analysis and yields of any EPS start with the isolation of pure polymers. In this regard, one of the most important issues is to avoid EPS contamination with components from microbial culture medium, usually mannan-containing yeast extract. Different EPS isolation protocols have been reported all of them including: (i) cell removal by centrifugation or filtration, (ii) polymer precipitation from the cell-free supernatant by the addition of chilled ethanol or acetone (the amounts needed to achieve the recovery of the EPS depended on the type of polymer released; two or three volumes are usually used), (iii) dialysis and drying of the precipitated polymer, and eventually (iv), a new reprecipitation and dialysis step (Ruas-Madiedo and de los Reyes-Gavilán, 2005). Purification of EPS can also include membrane-filtration, anion-exchange and/or gel permeation chromatography (Sanz and Martinez-Castro, 2007). In general, EPS isolation from high-protein content culture media (e.g., dairy products), proteins are typically removed by (i) precipitation with trichloroacetic acid, (ii) hydrolysis with proteases, (iii) or a combination of both.

Exopolysaccharides concentration is estimated as neutral carbohydrate content usually determined by the phenol–sulphuric acid method (Dubois et al., 1956) or by weighting the polymer dry matter (Vaningelgem et al., 2004). In addition, EPS concentration can be determined by means of high-performance size exclusion chromatography coupled with refractive index (RI) detection (HPSEC-RI); EPS concentration is calculated by the integration of the RI signal using calibration curves obtained with known MW dextrans (Sánchez et al., 2006).

Recently, Polak-Berecka et al. (2013) compared EPS yields using different extraction procedures. The authors found that a sample heat treatment as first step in the EPS isolation is critical for its complete recovery, especially when CPS or EPS-degrading enzymes are present. In addition, they assumed that centrifugal forces over 10,000 × *g* are too high being likely that part of the EPS is discarded together with the cell pellet. Considering the precipitation step, better results when prolonging the ethanol incubation (24 h at 4°C) under stirring were obtained. Goh et al. (2005) found that 70% (v/v) was sufficient to precipitate all dextran (2 × 10^6^ Da) present in a sample while centrifugation regimes after the ethanol precipitation step should last 30–40 min at 27,000–28,000 × *g*.

Capsular polysaccharides can be visualized by light microscopy after negative staining with Indian ink as described by Mozzi et al. (1995); thus, a white polysaccharide layer surrounding the cells can be observed. In addition, Malang et al. (2015) used crystal violet to stain the cells blue facilitating the differentiation of CPS negative cells on dark background. CPS isolation can be done by harvesting 24 h-cells, washing and resuspending them in 1/10 of the initial volume. Then, cell suspensions are heated at 90 °C for 15 min to detach cell bound polysaccharides (Mende et al., 2013). Cells are removed by centrifugation (15 min, 12,000 × *g*) and CPS are isolated from supernatants as described earlier for EPS.

As mentioned before, the major improvements in EPS characterization have been done related to techniques used for MW determination. Polymer MW was measuread based on the retention time of the polysaccharide eluted by HPSEC-RI; later, an increased use of HPSEC-MALLS was observed (Picton et al., 2000). In a higher degree of specificity or complexity, field flow fractionation (FFF) and hydrodynamic chromatography (HDC) can be used for determining the average MW of ultrahigh-MM polysaccharides (Cave et al., 2009; Isenberg et al., 2010; Galle et al., 2012b). Moreover, asymmetrical flow field fractionation (AF4) was successfully applied for the separation of starch-like glucans. Both, HPSEC and AF4, coupled with multiple inline detection of scattering intensities and mass profiles, provide distributions of apparent MW and radius of gyration with respect to the separated fractions (Rolland-Sabate et al., 2007; Juna et al., 2011).

The EPS monomer composition can be determined by total acid hydrolysis followed by monomer detection using high performance anion exchange chromatography (HPAEC) with pulsed amperometric detection (PAD; Cataldi et al., 2000). Alternatively, methanolysis and pertrimethylsilylation provide samples that can be analyzed by gas chromatography (GC). In addition, the monosaccharide analysis is used to determine the carbohydrate content, verifying the purity of the sample. The D or L configuration of the monosaccharide residues can be established by GC of the corresponding (-) 2-butyl glycosides (Gerwig et al., 1979). The linkage pattern of the monosaccharide constituents is determined after methylation of all free hydroxyl groups, followed by polymer hydrolysis, and further reduction of the monomers to alditols by sodium borodeuteride. Subsequent acetylation provides deuterated partially methylated alditol acetates that are analyzed by GC coupled with EI-MS (Ciucanu and Kerek, 1984). The percentages (ratio) of the terminal, internal, and branched glucose units as determined by the methylation analysis provides an idea of the polymer structure. Additional information about the structural features of the EPS can be obtained by ^1^H and ^13^C NMR spectroscopy. High-resolution NMR spectroscopy is the most powerful method for the unambiguous identification of carbohydrate chains (Damager et al., 2010). This method provides information of the type of constituent monosaccharides, ring size, and anomeric configuration, and the position of glycosidic linkages.

## Role of EPS-Producing LAB and their Polymers

Generally, LAB divert a small percentage of their sugar substrates toward the EPS synthesis, whose main biological function *in vivo* has not been exactly established. However, it has been reported that EPS may help to protect bacterial cells against harsh environmental conditions (i.e., desiccation, heat or acidic stresses, phage attack) and the presence of adverse compounds (i.e., antibiotics, human gastric and pancreatic enzymes, bile salts; Ozturk et al., 2009; Stack et al., 2010; Lebeer et al., 2011). In addition, EPS are involved in biofilm formation, host–pathogen interactions, and cellular recognition (Kumar et al., 2007; Gänzle and Schwab, 2009; Chapot-Chartier et al., 2011). Moreover, it has been claimed that EPS may play a role in the adhesion to surfaces such as eukaryotic cells (plants, human intestinal cells) and in the host immune system modulation (Russo et al., 2012; Salazar et al., 2015).

In biofilm formation, EPS production enhances local accumulation of microbes enmeshing them in the insoluble biofilm matrix (Koo et al., 2013). Dental plaque represents the best-known example of microbial biofilm whose formation begins with the adhesion of oral bacteria to the tooth surface. In the complex microbiome, *S. mutans* is the major EPS producer when sucrose and starch are present in the diet, favoring the *in situ* polymer (glucans and fructans) biosynthesis (Paes Leme et al., 2006; Bowen and Koo, 2011).

Krinos et al. (2001) demonstrated that like CPS, EPS can affect the surface antigenicity of different strains resulting in maintenance or elimination of specific strains in the gut ecological niche (Ruas-Madiedo et al., 2009b; Pessione, 2012). Welman and Maddox (2003) stated that EPS may create biofilms aiding in the colonization of the gut by increasing the gut-transit time of LAB. On the other hand, some EPS-producing LAB involved in alcoholic beverage spoilage causing ‘ropy’ textural alterations have been shown to be resistant to lysozyme treatment (Coulon et al., 2012).

Other studies failed to reveal a significant phage resistant phenotype in EPS and CPS producer strains (Deveau et al., 2002; Rodriguez et al., 2008); in contrast, McCabe et al. (2015) recently reported on the targeted recognition of *Lactococcus lactis* phages to their polysaccharide receptors.

In addition, it has been hypothesized that EPS may play a role as extracellular energy/carbon reserve; however, most EPS producer species lack the genes involved in their own EPS degradation (Badel et al., 2011). EPS could be useful as carbon reserve in the case of sintrophyc/symbiotic life with other bacteria, considering the overall population. Thus, glucans and fructans formed by oral streptococci and lactobacilli have major influences on the formation of dental plaque since they are involved in adherence of bacteria to each other and to the tooth surface serving as extracellular energy reserves to the non-lactic biota (Russell, 1994; Colby et al., 1995). However, Russo et al. (2012) observed a positive effect on the growth of *L. plantarum* WCFS1 and *L. acidophilus* NCFM in a glucose-containing chemically defined medium by the β-D-glucan isolated from *P. parvulus* 2.6, although the EPS alone did not serve as carbon source.

Interestingly, while EPS are synthesized by certain LAB species (i.e., *S. mutans*) under quorum-sensing control (related with biofilm formation and adhesion to solid surfaces; Kumar et al., 2007; He et al., 2015), in *L. reuteri* strains EPS formation has been demonstrated to be induced by environmental stress (Hüfner et al., 2008).

## Applications of EPS from LAB in the Food Industry

Exopolysaccharides from LAB have received special attention as valuable compounds because of their potential economic applications that include natural, safe-food additives or natural functional food ingredients increasing the possibility to replace or reduce the use of external hydrocolloids (Giraffa, 2004; Tieking et al., 2005a; Leemhuis et al., 2013a).

The large diversity of LAB species frequently isolated from raw materials and traditionally fermented foods (Van der Meulen et al., 2007; Endo et al., 2009; Robert et al., 2009; Ayeni et al., 2011; Shukla and Goyal, 2011; Di Cagno et al., 2013) has been the source of strains with interesting functionalities. In this sense, a wide diversity of EPS and genes encoding biosynthetic enzymes from naturally occurring LAB in fermented foods and beverages have been extensively studied for their role in the physicochemical (viscosifying, stabilizing, or water-binding capacities) and sensorial (palatability) characteristics in the final food products. A remarkable structural diversity of EPS produced by *Lactobacillus, Leuconostoc*, and *Weissella* strains has been reported (Uzochukwu et al., 2001; Olivares-Illana et al., 2003; Di Cagno et al., 2006; Mozzi et al., 2006; van Hijum et al., 2006; Van der Meulen et al., 2007; Bounaix et al., 2010a,b; Amari et al., 2012; Dimopoulou et al., 2014; Grosu-Tudor and Zamfir, 2014). With exception of dextran synthesized by *Leuc. mesenteroides*, EPS from LAB have not yet been commercially exploited as food additive due to their low yields (Monsan et al., 2001); nonetheless, the structural characteristics of EPS and the GRAS status of most of the LAB EPS producer strains allow considering the *in situ* production of texturing and/or biologically active EPS (Badel et al., 2011).

### Fermented Milks and Beverages

The use of EPS-producing cultures in the elaboration of fermented milks and beverages has been applied to reduce the amount of added milk solids, to improve the viscosity, texture, stability, and mouthfeel of the final products as well as to avoid syneresis (whey separation) during fermentation or upon storage (De Vuyst et al., 2001). Rheological problems like low viscosity, gel fracture or high syneresis are frequently encountered during fermented milk manufacture and can be solved by using EPS-producing LAB strains.

The contribution of the HePS to structure/function relationships is very complex. It has been postulated for example, that for high intrinsic viscosities stiffer chains, as the case of β-(1,4) linkages, are required, which in turn lead to higher consistency of the EPS solutions. In addition, the degree of branching may contribute to the stiffness of the polymer and while the complexity of the primary structure (namely size, monomer composition, and side groups, α- and β-linkages, branching) influence the viscosity of EPS solutions (Tuinier et al., 2001). As no clear-cut relationships of EPS yields and functional properties exist, a rational selection of the EPS-producing LAB strains for the production of yogurt and other fermented milks should be considered to increase the viscosity and texture of the final product through *in situ* EPS production. This was the case for the yogurt *S. thermophilus* EPS-producing strains (Vaningelgem et al., 2004).

Certain fermented beverages owe their organoleptic and sensorial characteristics to the presence of LAB and the *in situ* EPS production during the elaboration of the fermented drinks as *kefir* and *Pulque* (**Table 2**).

**Table 2:** Fermented beverages and foods containing EPS-producing LAB and effect of their polymers on their rheological and technological properties.

Fermented foods and beverages	EPS-producing microorganisms	Sensorial attributes	Reference
**Fermented beverages**			
Yogurt	*S. thermophilus; L. delbrueckii* subsp. *bulgaricus*	Increase in viscosity and texture improvement of fermented milks and beverages	De Vuyst et al., 2003; Vaningelgem et al., 2004
Kefir	*L. kefiranofaciens*		Maeda et al., 2004; Wang et al., 2008
Mexican *Pulque*	*Leuc. mesenteroides*		Escalante et al., 2008
**Cheeses**			
Low-fat Italian *Cacciotta* type	*S. thermophilus*	Pleasant to taste and chew, flavor, overall acceptability	Di Cagno et al., 2014
Egyptian *Karish*		Enhanced acceptability, spreadability, and creaminess	Hassan et al., 2004
			
Reduced-fat	*S. thermophilus* TM11	Increased moisture content and high yield	Wang et al., 2012
Mexican *Chihuahua*	EPS-producing starter culture	Improved cheese yield and texture	Trancoso-Reyes et al., 2014
Reduced-fat Cheddar		Improved body and texture	Agrawal and Hassan, 2008
**Fermented Breads**			
Gluten-free breads	*W. cibaria; W. confusa; Leuc. mesenteroides; L. sanfranciscensis*	Enhanced texture and quality	Schwab et al., 2008; Galle et al., 2012a; Rühmkorf et al., 2012; Wolter et al., 2014a,b
Gluten-free sorghum	*L. casei FUA3185* and *FUA3186, L. buchneri FUA3154*	Improved rheology of sorghum sourdoughs	Galle et al., 2011
Wheat	*Leuc. lactis*, *L. curvatus*	Improved viscoelasticity and quality	Palomba et al., 2012

*Kefir* is a viscous, slightly carbonated and alcoholic dairy beverage, traditionally consumed in Eastern European countries. Kefir is produced by bacteria and yeasts contained in kefir grains that have unique taste and properties. During fermentation, peptides, and EPS showing bioactive characteristics are formed. Kefiran is a water-soluble EPS produced in kefir grains, which consist of a complex population of LAB and yeasts firmly embedded (Cheirsilp et al., 2003). The principal kefiran producers in kefir grains is *L. kefiranofaciens* and several other unidentified species of lactobacilli. Kefiran is a branched glucogalactan composed of hexa- or heptasaccharide repeating structure with almost equal amounts of glucose and galactose (Maeda et al., 2004; Medrano et al., 2009). The MW of kefiran obtained from kefir grain has been reported as higher than 10^7^ Da by Piermaria et al. (2008) while Maeda et al. (2004) found a MW value of 7.6 × 10^5^ when culturing the single strain *L. kefiranofaciens* WT-2B(T) isolated from kefir grain in liquid medium containing a rice hydrolysate.

*Pulque* is a traditional Mexican, non-distilled alcoholic fermented beverage consumed mainly in Central Mexico. It is obtained from the fermentation of a fresh sap known as *aguamiel*, which is extracted from different Agave species. Freshly collected *aguamiel* is deposited in open containers where previously fermented *pulque* acts as seed for a new batch. Fermentation time varies from a few hours to several days. The viscosity resultant from EPS synthesis and the alcoholic content of the beverage are features used to determine the extent of fermentation and the sensorial properties of *pulque* (Escalante et al., 2008). Among LAB, *Leuc. mesenteroides* has been traditionally considered one of the most important microorganisms during *pulque* fermentation due to its ability to synthesize dextrans from sucrose present in *aguamiel* and *pulque*. The structure of an EPS produced by the strain *Leuc. mesenteroides* IBT-PQ isolated from *pulque* revealed the presence of a soluble linear dextran with glucose molecules linked by α-(1,6) bonds with branching from α-(1,3) bonds in a 4:1 ratio, respectively. A great LAB diversity in *aguamiel* and *pulque* samples from different geographical origins, composed mainly of *Lactobacillus* and *Leuconostoc* species, has been reported (Escalante et al., 2004). *Leuc. citreum* and *L. kimchii* were reported to be the most abundant LAB species present in *aguamiel* during the early stages of *pulque* fermentation (Escalante et al., 2008).

### Cheeses

It has been reported that EPS-producing LAB improve the sensory attributes of various cheeses, especially those reduced- and low-fat varieties where the decrease in the fat content negatively affects the textural and meltability properties of cheese (**Table 2**). In this sense, the application of EPS-producing strains for improving the texture and technical properties of reduced-fat cheese has been very promising. EPS produced by non-ropy strains have drawn the attention of the dairy industry since their ability to produce CPS and EPS could improve the texture of dairy products without causing the undesirable slippery mouthfeel produced by the ropy strains (Hassan, 2008).

Recently, Di Cagno et al. (2014) used an EPS-producing *S. thermophilus* strain to produce low-fat Italian *Caciotta*-type cheese. The sensory attributes of cheeses containing the EPS-producing strain were pleasant to taste and to chew, showed intensity of flavor, and overall acceptability. Based on these observations, 14-day ripened low-fat *Caciotta*-type cheese had promising features to be further exploited as a suitable alternative to the full-fat variant.

Costa et al. (2010) found that reduced-fat *Cheddar* cheese made with an EPS-producing *Lactococcus lactis* strain displayed an 8.2% increase in yield (per 100 kg of milk), 9.5% increase in moisture content, and increase in water activity and water desorption rate. Interestingly, the presence of EPS did not negatively affect the flavor profile of the cheese.

Agrawal and Hassan (2008) studied the technological characteristics of reduced-fat *Cheddar* cheese made with ultrafiltrated (UF) milk and an EPS-producing culture. The authors observed that UF did not affect the hardness, cohesiveness, adhesiveness, chewiness, and gumminess of the EPS-containing cheese while the springiness of the EPS-containing cheese made from UF milk was much lower than that of the non-EPS cheeses. Texture of the EPS-negative cheese was more affected by UF than the EPS-positive cheese. While UF increased the elastic modulus in the 6-month old EPS-positive cheeses, higher body, and texture scores were given to EPS-positive cheeses than to EPS-negative ones.

Hassan et al. (2004) reported that the inclusion of EPS-producing cultures in the traditional Egyptian cheese *Karish* enhanced consumer acceptability by improving their spreadability and creaminess. This product is conventionally produced by adding fat, sugar, protein or stabilizers like pectin, starch, alginate or gelatin; thus, the addition of EPS cultures could be an interesting and viable alternative to the use of exogenous polysaccharides. Moreover, EPS from LAB may prolong the retention time of the milk product in the mouth, enhancing its delicacy.

More recently, Trancoso-Reyes et al. (2014) observed that the addition of EPS alone improved the Mexican *Chihuahua* cheese yield by increasing water and fat retention, causing, however, a negative effect on the texture and flavor of the cheese. When authors used the EPS-producing bacteria in combination with a phospholipase-A1 (PL-A1)-producing strain, an improvement on cheese yield, moisture, and fat content was observed. The cheeses showing the best flavor and texture were those manufactured with PL-A1 and with the combination of PL-A1 and the EPS-producing culture.

In addition, the EPS-producing strain *S. thermophilus* TM11 was evaluated for the production of reduced-fat cheese using reconstituted milk powder (Wang et al., 2012). The physicochemical analysis of fresh and stored cheeses showed that this strain slightly increased moisture content resulting in cheese with higher yield and lower protein content compared to the direct acidified cheese.

In summary, EPS provide functions that benefit reduced-fat cheeses since they bind water and increase the moisture in the non-fat portion, interfere with protein–protein interactions, reduce the rigidity of the protein network, and increase viscosity of the serum phase (Hassan, 2008).

### Fermented Breads

Grinding of cereals and addition of water results in the formation of a dough that after some time will turn into a sourdough with characteristic acidic taste, aroma and increased volume due to gas formation. Cereal fermentation goes back to ancient times as one of the early microbial processes employed by man leading to the use of sourdough for bread-making (Hammes and Gänzle, 1997). The sourdough process was rediscovered because of the effects on the sensory, structural, nutritional and shelf life properties of leavened baked goods (Arendt et al., 2007; Gobbetti et al., 2014). In sourdough fermentation LAB and yeast communities are involved; while LAB dominate the microbial community and are responsible for acid production, yeasts are responsible for dough leavening. LAB are involved in both decreasing the α-amylase activity and improving dough texture. In addittion, sourdough LAB may synthesize a large variety of EPS through GS activity. Since these bacteria are used as starters in cereal fermentations, these polymers are available for food applications through the *in situ* biosynthesis during processing (Tieking and Gänzle, 2005; Bounaix et al., 2009).

Lactic acid bacteria able to produce HoPS are already applied in the elaboration of conventional bread (Lacaze et al., 2007); however, their use is more promising in gluten-free baking since EPS can potentially act as hydrocolloids improving their rheological properties (Schwab et al., 2008; Galle et al., 2012a; Rühmkorf et al., 2012).

It has been reported that microbial *in situ* production of EPS during sourdough fermentation is more effective than the addition of comparable amounts of EPS to the bread formulation. The ability to produce HoPS by LAB strains during sourdough fermentation depends on the metabolic activity of the fermentation microbiota (Gänzle et al., 2007), and contributes to the sourdough ability to influence bread quality (Katina et al., 2009; Galle et al., 2012a; Palomba et al., 2012). More recently, Gobbetti et al. (2014) have observed that the *in situ* EPS formation was responsible for the significant decrease of dough strength and elasticity; however, dextran produced showed the best shelf life improvement.

Dextran production from sucrose is a phenotypic identification characteristic of the *W. confusa* and *W. cibaria* species. *Weisella* strains have been used for *in situ* dextran and GOS synthesis to improve the texture and quality of wheat and gluten-free breads (Schwab et al., 2008; Katina et al., 2009; Galle et al., 2010, 2012a; Wolter et al., 2014a,b). In this sense, high MW dextrans are used in sourdough baking to produce good quality bread (Maina et al., 2011). Dextrans have displayed prebiotic potential (Olano-Martin et al., 2000) and have been already approved by the European Commission to be used as additive in bakery products.

Exopolysaccharides produced by certain lactobacilli, such as the β-(2,6) levan synthesized by the sourdough strain *L. sanfranciscensis*, positively influence bread textural properties by facilitating water absorption, softening the gluten content of dough, improving the structure build-up, retarding bread staling and prolonging shelf life (Tieking and Gänzle, 2005).

In the traditional Italian sweet bread panettone, dextran produced by a *Leuc. mesenteroides* strain was responsible for the long storage stability (Decock and Cappelle, 2005). In this product, dextran production was optimized by transferring the doughs seven times. In sweet wheat milk bread, a remarkable reduction in bread firmness along the storage period was obtained (Lacaze et al., 2007).

As mentioned above, EPS isolated from sourdough include mainly HoPS; however, Van der Meulen et al. (2007) reported the production of HePS from a sourdough isolate strain of *L. curvatus*, which synthesized an EPS composed of galactosamine, galactose, and glucose in a ratio of 2:3:1, respectively. In addition, Galle et al. (2011) have investigated the influence of HePS-producing LAB in sourdough fermentation and found that the use of this type of EPS positively affected the rheological properties of sorghum sourdough, expanding the diversity of EPS and the variety of cultures used for baking.

## Concluding Remarks

Exopolysaccharides-producing LAB hold great potential for the functional food sector either as starter and adjunct cultures or as *in situ* supplier of bioactive polymers with positive impact on the rheology of fermented products or on the human health. The increased knowledge concerning the enzymes involved in HoPS synthesis and their regulation open new possibilities for their use to improve the texture of sucrose-supplemented products. Most of the research done on EPS during the last decade was focused on the evaluation of HoPS structures as well as the novel functionalities of HePS. In this concern, the requirement of specific methodologies (FTIR, NMR, MALLS, FFF, AF4, etc.) for the EPS structural analysis as well as the *ex vivo* assays (cell tissues) to determine their biological activity was essential. Nevertheless, the structure/function relationships of EPS should be *in situ* proved in foods or other matrices of interest. The improvement of EPS production to reduce their industrial costs and the design of tailor-made EPS with desired/specific functionalities remain challenging.

## Conflict of Interest Statement

The Associate Editor Julia Ines Fariña declares that, despite being affiliated with the same institution as authors María Inés Torino, Graciela Font de Valdez and Fernanda Mozzi, the review process was handled objectively. The authors declare that the research was conducted in the absence of any commercial or financial relationships that could be construed as a potential conflict of interest.
